# Similarities and Differences Between Bullying and Sexual Harassment in Schools: A Social-Ecological Review of Risk and Protective Factors

**DOI:** 10.3390/bs15010061

**Published:** 2025-01-13

**Authors:** Zehra Sahin-Ilkorkor, Sarah Jane Brubaker

**Affiliations:** L. Douglas Wilder School of Government and Public Affairs, Virginia Commonwealth University, Richmond, VA 23284, USA; sbrubaker@vcu.edu

**Keywords:** bullying, sexual harassment, social-ecological model, school, youth violence, peer victimization, community stakeholders

## Abstract

In this conceptual article, the authors provide a narrative review of literature on bullying and sexual harassment in K-12 schools framed through a comparative analysis of risk and protective factors for both forms of violence across the social-ecological spectrum. We find that a greater number of studies of both forms of violence focus on student and microsystem-level factors rather than on higher levels of the ecosystem including school boards, neighborhoods, and broader cultural norms. In addition, the research overwhelmingly identifies more risk factors than protective factors. Finally, we find more similarities than differences in risk and protective factors between the two forms of school-based violence. We identify implications of the findings for theory, research, and policy and suggest that preventing any form of harassment at school will benefit not only students but the entire school community. We argue that the causes of school-based harassment are complex and require comprehensive prevention, intervention, and response approaches that address shared risk and protective factors, particularly those at the community and mesosystem, exosystem, and macrosystem levels. Ultimately, we argue that all community stakeholders must be committed to and engaged in these endeavors for them to be successful.

## 1. Introduction

Bullying and sexual harassment are two forms of aggression that have been recognized as significant problems in schools and are associated with a variety of externalizing, internalizing, and risky health behaviors (Doty et al., 2017). According to a uniform definition developed by the U.S. Department of Education, U.S. Centers for Disease Control and Prevention, and the Health Resources and Service Administration, “[b]ullying is any unwanted aggressive behavior(s) by another youth or group of youths who are not siblings or current dating partners that involves an observed or perceived power imbalance and is repeated multiple times or is highly likely to be repeated” (Gladden et al., 2014, p. 7). The U.S. Department of Education’s definition of sexual harassment is behavior that “is sexual in nature; is unwelcome; and denies or limits a student’s ability to participate in or benefit from a school’s education program” (U.S. Department of Education, Office for Civil Rights, 2008, p. 3). These two phenomena occur at varying levels, with most prevalence estimates of bullying falling between 8 and 32 percent (Cascardi et al., 2018) and prevalence estimates of sexual harassment in schools ranging from 37 to 56 percent for girls and from 21 to 40 percent for boys (Ormerod et al., 2023).

Research on bullying and sexual harassment has historically followed distinct trajectories, involving different theoretical backgrounds, disciplines, empirical findings, policies, and approaches to intervention and prevention (Doty et al., 2017; Gruber & Fineran, 2016). However, studying one form of violence in isolation may hinder our ability to comprehend why some people are more vulnerable to being victims and/or perpetrators, and how to better prevent and intervene in violent incidents (Grych & Swan, 2012). It can also result in the continual reinvention of theoretical and methodological wheels as well as the overlooking of significant shared risks and consequences (Grych & Swan, 2012). Hence, some researchers have called for efforts to collaborate and to understand the interconnections across multiple forms of violence (Grych & Swan, 2012; Smokowski & Evans, 2019). Furthermore, the Centers for Disease Control and Prevention’s Division of Violence Prevention started a five-year mission in 2016 to understand the connections between multiple forms of violence in an effort to bring together different bodies of violence research (Smokowski & Evans, 2019).

Bullying and sexual harassment have most often been considered distinct entities and studied in isolation even though they are interconnected. The existing literature examining the links between bullying and sexual harassment mainly focuses on the development pathways of how one precedes the other and whether one is a risk factor for the other. A growing body of longitudinal and cross-sectional studies indicates that bullying is a risk factor for other forms of violence such that bullying perpetration positively predicts sexual harassment perpetration (Humphrey & Vaillancourt, 2020; DeSouza & Ribeiro, 2005; Pepler et al., 2006; Pellegrini, 2001), sexual violence perpetration (Espelage et al., 2015), and dating violence perpetration and/or victimization (Ellis & Wolfe, 2015; Foshee et al., 2014; Cutbush et al., 2016) at a later time point. However, commonalities and distinctions between these two phenomena have not been reviewed, and little is known about the risk factors or protective factors that can be present for both bullying and sexual harassment at various levels of the ecological context.

To achieve a more thorough understanding of the topography of violence in schools, this article provides a narrative review of the separate bodies of research on bullying and sexual harassment in K-12 school contexts. Through this comprehensive review, this article aims to answer the following research question: What are the shared risk and protective factors that alter the likelihood of perpetration or victimization of bullying and/or sexual harassment? Using a social-ecological approach, we examine how relationships with parents, peers, teachers, and other stakeholders in the school community may function as risk and protective factors against bullying and sexual harassment. We also analyze how hostile and violence-supportive climates might cause the overlapping experiences of perpetrators, victims, and bystanders, across all stakeholders and community members.

Understanding the interconnections, commonalities, and distinctions between bullying and sexual harassment has significant implications for research and policy. First, comparing bullying and sexual harassment will help us understand the shared risk and protective factors in the separate bodies of literature. Hence, schools can adopt prevention and intervention programs that are responsive to the overlapping components of each form of violence. This integrated prevention approach allows schools to address both incidents jointly, maximize benefits by focusing on shared risk factors, and reduce possible redundancy and duplication (Cascardi et al., 2018). Also, this integrated approach might result in more significant reductions in each respective aggressive behavior (Cascardi et al., 2018). Second, comparing bullying and sexual harassment will allow us to identify distinct risk and protective factors that are investigated in only one form of violence but may have implications for the other. According to Stein (2003, p. 794), the majority of bullying researchers are not familiar with disciplines other than psychology, and “the field of bullying research will be greatly enhanced once it builds upon researchers from other fields who have long studied the arena of gender violence and sexual harassment in schools”. Thus, drawing comparisons between bullying and sexual harassment can bring disciplines together and contribute to separate bodies of literature. Third, a comparison of bullying and sexual harassment through the social-ecological lens can help us better understand the role of various stakeholders in school violence and develop policies that can target the most influential stakeholders.

The organization of this article is as follows. First, we provide an overview of the conceptual framework that guides our method and analysis. Next, we describe the methods we employed to select and analyze the literature. In the Results section, we summarize findings from the literature on the risk and protective factors of bullying and sexual harassment at each level of the social-ecological system, with a focus on similarities and differences between both forms of violence. The final section offers a discussion of the comparative findings across the ecological spectrum and implications of these findings for policy, practice, and future research directions in this area.

## 2. Social-Ecological Model

Researchers and practitioners across a range of disciplines utilize versions of the social-ecological model. Most attribute the model to Bronfenbrenner (1979), who posited that a child’s development is a product of both their biology and a series of systems that surround and influence the child. Researchers have applied this framework to a myriad of public health, educational, and other social problems because these approaches are comprehensive, and “multidimensional approaches to understanding human behavior are more inclusive than single-factor theories, and often identify important influential factors that those theories might miss” (Brubaker, 2019, p. 115).

Bronfenbrenner’s (1979) social-ecological model explains a child’s development by studying both the biological and genetic aspects of a child and the entire ecological system that surrounds the child. The social-ecological approach assumes the dynamic and multifaceted nature of human behavior and emphasizes the importance of considering individual factors, interactions between individuals and their environments, and interactions across systems (Brubaker, 2019). The model is illustrated by concentric or nested circles, each representing a system that influences the individual: microsystem, mesosystem, exosystem, macrosystem, and chronosystem. These levels are further defined and conceptualized by Allen et al. (2018) in the school setting and educational context as such: (i) Student—emotional stability, personal characteristics, academic motivation; (ii) Microsystem—teacher support, parent support, peer support; (iii) Mesosystem—school policies, extracurricular activities, staff professional development, rules, practices; (iv) Exosystem—shared whole-school vision, school board, neighborhood, extended family, broader; and (v) Macrosystem—history, social climate, government-driven educational reform and data collection, culture, legislation (Allen et al., 2018). We selected and reviewed previously published research and organized data gathered from the retrieved articles according to this specific framework and systems of the social-ecological model.

## 3. Methods

Literature reviews fall into one of two categories: systematic or narrative (Byrne, 2016). Narrative reviews (also known as non-systematic reviews or traditional reviews) can build themes according to broad reviews of previously published research using more flexible search methods and inclusion criteria (Green et al., 2006). In contrast, systematic reviews identify relevant sources via strict methodological inclusion and exclusion criteria based on a focused research question (Green et al., 2006). There are no recognized guidelines for narrative reviews, while systematic reviews require standards like the PRISMA (Preferred Reporting Items for Systematic Reviews and Meta-Analyses) (Ferrari, 2015; Green et al., 2006). Additionally, narrative reviews may or may not contain the Methods section, but doing so clarifies the main points of the narrative reviews (Green et al., 2006; Ferrari, 2015; Collins & Fauser, 2005). Even though systematic reviews are regarded as the gold standard for synthesizing evidence, “narrative reviews remain frequent within the literature, as they offer breadth of literature coverage and flexibility to deal with evolving knowledge and concepts” (Byrne, 2016, p. 2). 

This article adopted a narrative review because having a more flexible method for locating and synthesizing the available literature allowed us to present a broad perspective on the topic. We aimed to identify articles that addressed the risk and protective factors at different levels of society, particularly those that identified various stakeholders in the school community. Using general search terms could have resulted in studies that only addressed risk and protective factors at certain levels of society, whereas having many search terms encompassing all levels of society would have made the data extraction process less feasible. Unlike narrative reviews, systematic reviews require specific queries and search terms (Ferrari, 2015). Hence, this article adopted a narrative review that allowed us to reformulate our search terms by taking into account the findings of the preliminary screening. This review examined the research on bullying, sexual harassment, or both forms of violence in the school setting. After adopting a broad focus that included prevalence rates and conceptual definitions, we carried out more targeted searches on risk and protective factors, paying particular attention to stakeholder involvement.

We applied the social-ecological approach to our selection of literature (e.g., Hong & Espelage, 2012; Patton et al., 2013) to identify risks and protective factors at different levels of society. We carried out searches through Google Scholar because it updates its database more often than other relevant search engines, provides data on the number of citations, and includes the “cited by” option of a specific article. This helped us identify the latest research and access to additional research related to the article that was retrieved. We first identified articles that addressed both forms of violence by using the search terms “bullying” AND “sexual harassment”. We further included search terms “risk factors”, and “protective factors” paired with bullying and/or sexual harassment, secondary schools or K-12 schools. Based on the results of the screening, we further combined these search terms with the factors mentioned at higher levels of society, specifically “school prevention policies”, “media”, “cultural factors”, and “cultural norms” (Allen et al., 2018). To fully capture the involvement of stakeholders in the school community, we put particular emphasis on the impacts and roles of parents, peers, teachers, and school staff on bullying and/or sexual harassment. Furthermore, we located additional articles through hand searches of the references of articles that were retrieved.

We focused on the last decade of research but did not restrict our search by any particular time frame to give a thorough review and not exclude highly cited publications. We also did not limit the geographic concentration of our search, but the majority of articles come from the US context, while few articles focused on other countries including Canada, Finland, Switzerland, South Korea, Brazil, and Chile. We included English-language peer-reviewed articles and book chapters, and we also cited selected government reports while discussing prevalence rates and school policies. We relied on systematic reviews, meta analyses, and narrative reviews on this topic, when available. The results section of this narrative review includes 91 peer-reviewed articles, 7 books or book chapters, and 6 government reports. The literature on bullying and sexual harassment that took place outside of K-12 schools such as on college campuses and in the workplace were excluded from our review. 

## 4. Results

For each level of the social-ecological model, we summarize similarities and differences between bullying and sexual harassment. In keeping with the focus of the model, we identify factors at each level that contribute to the prevalence, likelihood, and experiences of school-based violence, including both risk and protective factors.

### 4.1. Student

In this section, we address similarities and differences between student-level factors. We added demographic characteristics and identity categories to Allen et al.’ (2018) categories of personal characteristics, emotional stability, and academic motivation that have been identified as risk and protective factors for bullying and sexual harassment victimization and perpetration.

#### 4.1.1. Age and Grade Level

One difference in risk factors that we noted between school-based bullying and sexual harassment is the prevalence of each by grade level. For example, much of the existing research has found that the frequency of bullying perpetration rises in middle school and declines in high school (Pepler et al., 2006; Kljakovic & Hunt, 2016; Hong & Espelage, 2012). Pellegrini (2002) and Pepler et al. (2006), for example, argue that bullying behavior increases when students make this school transition because aggression is motivated by adolescents’ exploration of new social roles and their pursuit of status among peers in their new school settings.

In a comparative study, younger students (8th grade) have been found to be at greater risk of bullying victimization or perpetration, whereas older students (9–11 grades) were at greater risk of sexual harassment perpetration (Doty et al., 2017). Sexual harassment victimization, on the other hand, did not vary significantly by age (Doty et al., 2017). Espelage et al. (2016) have noted that most of the studies of school-based sexual harassment have focused on high school, so the lack of research in middle schools may contribute to the impression that it happens more often in high schools.

Related to age, similar between bullying and sexual harassment are the developmental factors that contribute to the risk for experiencing both forms of violence at school (Holmqvist Gattario & Lunde, 2023; Skoog et al., 2023). For example, adolescence involves a rapid period of psychological, emotional, social, and physical changes as young people experience sexual maturation and transition into adulthood. Consistent with developmental expectations, Pepler et al. (2006) found that sexual harassment rose in early adolescence and was associated with pubertal development but lowered in later high school. Craig et al. (2001) studied sexual harassment among middle school students and reported that 28% of those who had already experienced sexual harassment had experienced early onset puberty, while 18% had experienced puberty on time and 10% had experienced late onset puberty. Additionally, Turner-Moore et al. (2022) found that boys were more likely to perpetuate sexualized bullying during adolescence, while girls were more likely to be victimized.

#### 4.1.2. Social Identities

A similar risk factor, revealed in recent literature on both bias-based bullying and sexual harassment, is students’ marginalized social identities based on race, ethnicity, religion, gender, sexual orientation, and disability, which motivate violence perpetration. Research consistently suggests that youth with disabilities, LGBTQ youth, overweight and obese youth, and low-income youth are at higher risk of bullying victimization (Gruber & Fineran, 2008; Hong & Espelage, 2012).

##### Religion and Ethnicity

Research on both forms of violence have reported mixed findings on the influence of religious, ethnic, and race identities, but we share some of the major findings here. Limited research on religious affiliation suggests that religious values and affiliation can be a risk factor for bullying victimization as well as a protective factor against bullying (Hong & Espelage, 2012). A systematic review by Sapouna et al. (2023) finds that racial/ethnic minority, immigrant, and refugee youth are at a higher risk of bullying victimization compared to non-minority groups. However, there are mixed findings in the literature concerning racial/ethnic minority status and bullying perpetration. A systematic review by Álvarez-García et al. (2015) find that racial/ethnic minorities are more likely than non-minority groups to bully others, whereas Barboza et al. (2009) find that White students are more likely than Black students or Asian students to bully others. On the other hand, Basile et al. (2020) reported that “white students had the highest prevalence of experiencing bullying victimization at school and electronic bullying. The prevalence of electronic bullying among Hispanic students was also significantly greater than the prevalence among black students” (Basile et al., 2020, p. 4).

Similarly, limited research has examined differences by race and ethnicity in experiences of sexual harassment in schools (Skoog et al., 2023), and the few studies that exist show mixed results (Espelage et al., 2016). Espelage et al. (2016), for example, found that White middle school students reported more sexual harassment victimization than African American students. African American girls, however, were the group to report the highest rates of physical sexual assault, followed by African American boys. Some studies (e.g., Wilmot et al., 2021; Harris & Kruger, 2023) suggest that Black girls experience more direct forms of sexual harassment than White girls, from both peers and teachers. Because of stereotypes of Black girls’ strength, independence, and sexuality, however, teachers do not often label the behaviors as sexual harassment or provide support.

##### Gender

There are also mixed findings in the literature about the role of gender in bullying involvement, and they all focus on risk factors. For example, the National Center for Education Statistics (2024) found that girls aged 12–18 experienced more bullying victimization than boys overall, in the form of rumors, being made fun of and being called names, and being excluded. Boys experienced more bullying in the form of physical harassment such as being pushed, kicked, shoved, or spat on. Basile et al. (2020) also found that girls were significantly more likely than boys to experience bullying. Some studies report that boys, in general, are more likely than girls to bully others (e.g., Gruber & Fineran, 2008; Nansel et al., 2001; Baldry, 2003); some showed that girls are more likely to be victims of relational bullying, while boys are more likely to experience physical and verbal bullying (e.g., Olweus, 1993); others showed that girls are less likely than boys to become perpetrators or victims of bullying (e.g., Lam et al., 2015; Cook et al., 2010); and some others showed that gender is not a significant predictor of bullying perpetration or victimization (e.g., Barboza et al., 2009; Kljakovic & Hunt, 2016).

Studies of sexual harassment generally find that girls experience more sexual harassment victimization than boys and that boys perpetrate more than girls (Espelage et al., 2016; Gruber & Fineran, 2008). However, there is a wide range in prevalence rates across studies, likely due to inconsistent measurement (Gruber & Fineran, 2008). According to Ormerod et al. (2023), researchers report that between 37 and 56% of girls and between 21 and 40% of boys experience sexual harassment in schools.

Some researchers find gender differences across various types of sexual harassment. For example, Rolfe and Schroeder (2020) found that boys perpetrate more verbal sexual harassment against both girls and boys, but prior dating experience increases the odds of girls’ verbal sexual harassment of boys. Boys have also been found to experience more same-sex harassment than girls, who are more likely to experience harassment from boys (Espelage et al., 2016). Brown et al. (2023) found that, “The most commonly self-reported type of SH [sexual harassment] experienced was someone making comments about or rating parts of their body, reported by 21.4% of students (12.5% of teen boys, 23.6% of teen girls… Taken together, gender differences emerged such that girls heard more comments made about their bodies and more unwelcome sexual jokes or comments made about them” (Brown et al., 2023, p. 1421).

Research also suggests that girls experience more negative consequences from sexual harassment victimization than do boys, including psychological and health problems (Gruber & Fineran, 2008). In a study in Finland, Kaltiala and Ellonen (2022) found that all forms of sexual harassment (gender harassment, unwelcome sexual attention, and sexual coercion) were reported more by adolescents with opposite sex and non-binary gender identity than by cisgender adolescents.

##### Sexual Orientation

Lesbian, gay, and bisexual (LGB) students experience higher rates than straight students of both forms of victimization (Basile et al., 2020; Gruber & Fineran, 2008; Johns et al., 2020; Smith et al., 2022), and they experience more negative consequences from victimization than their straight peers (Gruber & Fineran, 2008). Johns et al. (2020) found additional gender and race/ethnicity differences in violence victimization among LGB students. Specifically, among LGB students, males were more likely to feel unsafe at school and to report being threatened by a weapon, where female students were more likely to report being bullied both at school and online. Female LGB students also reported more sexual dating violence and forced sex than male students. Compared to White LGB students, Black and Hispanic LGB students were more likely to report feeling unsafe at school and being threatened by a weapon but were less likely to report bullying at school and online (Johns et al., 2020). Basile et al. (2020) found that female students, LGB students, and students not sure of their sexual identity consistently had the highest prevalence across all five of the violence victimization indicators “(1) having experienced physical dating violence, (2) having experienced sexual dating violence, (3) having experienced sexual violence by anyone, (4) having been bullied on school property, and (5) having been bullied electronically” (Brown et al., 2020, p. 3).

##### Disability

Students with disabilities are at increased risk of experiencing bullying (Rose et al., 2022; Rose & Gage, 2017). This involvement begins as early as preschool (Son et al., 2012) and persists over time (Rose & Gage, 2017). As a whole, students with disabilities are more than 1.5 times more likely to be victimized than their typically developing peers (Blake et al., 2012). Students with autism spectrum disorders or intellectual disabilities experience higher rates of victimization than their peers served under other disability categories (Symes & Humphrey, 2010; Twyman et al., 2010; Zablotsky et al., 2012).

Although research on sexual harassment experienced by children with disabilities in the U.S. is scant, there is reason to believe that there is a disproportionate rate of sexual harassment among this population. For example, children with disabilities are much more likely to experience sexual abuse at the hands of family members, peers, and caregivers (Mailhot Amborski et al., 2022). Researchers have noted the high rates of sexual harassment experienced by children with disabilities in other countries (see, for example, López et al., 2020; Peris, 2020). It is also likely that at least some of the bullying experienced by children with disabilities is gendered or sexualized. Researchers also agree that students with disabilities receive insufficient sex education and sexual harassment awareness training (Goli et al., 2022; Klebanov et al., 2024).

#### 4.1.3. Personality Characteristics & Emotional Stability

Individual-level factors have been associated with both forms of violence perpetration including high levels of anger, low self-esteem, pornography exposure, and homophobic, sexist, and traditional masculinity values (Leemis et al., 2019). For example, Foshee et al. (2016) found that adolescent’s depression, feelings of anger, anger reactivity, poor conflict management skills, and adolescent acceptance of sexual violence are all shared risk factors for bullying and sexual harassment perpetration. Research finds that the frequency of bullying perpetration is higher for both boys and girls who had high scores on general misconduct compared to those who had low scores on general misconduct (DeSouza & Ribeiro, 2005). The frequency of bullying perpetration is higher for boys who had low scores on benevolent sexism compared to those boys who had high scores on benevolent sexism (DeSouza & Ribeiro, 2005). Research also suggests that some personality characteristics (such as anger, poor impulse control, low empathy, delinquency, alcohol and substance use, conduct problems, a hostile worldview, and positive attitudes toward violence) are risk factors for bullying perpetration (Smokowski & Evans, 2019; Basile et al., 2009; Vassallo et al., 2014; Kljakovic & Hunt, 2016). Research consistently suggests that youth with depressive symptoms are at higher risk of bullying victimization (Hong & Espelage, 2012).

Young people who abuse substances are at great risk for sexual harassment victimization (Skoog et al., 2023), and those who have alcohol use and delinquency issues have a higher likelihood of sexual harassment perpetration and victimization (Fineran & Bolen, 2006). Skoog and Kapetanovic (2023) found concurrent and reciprocal effects between sexual harassment and emotional problems over time. Holmqvist Gattario and Lunde (2023) suggest that young people’s attitudes and norms regarding sexual harassment are important risk and protective factors. For example, Herrera Hernandez and Oswald (2023) found that “young adults’ tolerance for sexual harassment is positively associated with endorsement of rape myth acceptance and sexism” (Herrera Hernandez & Oswald, 2023, p. 1119). Rolfe and Schroeder (2020) found that “students with dating experience are more likely to be victims or perpetrators of verbal sexual harassment”, and that the relationship is stronger for female students (Rolfe & Schroeder, 2020, p. 17).

#### 4.1.4. Academic Motivation

Good academic/school performance is demonstrated as a protective factor for bullying victimization (Hemphill et al., 2014; Vassallo et al., 2014), and bullying perpetrators are more likely to perform poorly academically than other students (Cook et al., 2010). Although academic disengagement has been found to be associated with experiences of both bullying and sexual harassment (Government Accountability Office, 2021; Ormerod et al., 2023), researchers have not identified academic motivation as a protective factor for sexual harassment.

### 4.2. Microsystem

In this section, we examine students’ interpersonal relationships that have been identified as risk and protective factors related to bullying and sexual harassment, including strong ties to peers who engage in these behaviors and exposure to family violence. These microsystem factors include important stakeholders in terms of prevention practices and policies.

#### 4.2.1. Parents

Research suggests that witnessing parental/familial violence and negative parenting styles is a risk factor for both forms of violence. For example, a meta-analysis by Lereya et al. (2013) found that both bullying victims and perpetrators were more likely to witness negative parenting styles compared to those who were not involved in bullying either as a victim or perpetrator. A few studies suggest that exposure to inter-parental violence at home is a risk factor for both bullying perpetration (Cook et al., 2010; Baldry, 2003) and bullying victimization (Baldry, 2003), even though some studies do not find a significant association between exposure to parental conflict and bullying perpetration/victimization (Bauer et al., 2006; Leemis et al., 2019). Like bullying, experiences of sexual harassment have been shown to increase for young people who witness general violence (Fineran & Bolen, 2006) and intimate partner violence at home (Clear et al., 2014). Foshee et al. (2016), for example, found that “mother–adolescent discord, family conflict, low maternal monitoring, low mother–adolescent closeness, low family cohesion” were all shared risk factors across dating violence, bullying, and sexual harassment perpetration (Foshee et al., 2016, p. 2).

In addition to witnessing family violence, other family-related factors can be risk factors for both forms of violence. Wang et al. (2009), for example, found that “adolescents from more affluent families were less likely to be physical victims but more likely to be cyberbullying victims” (Wang et al., 2009, p. 372). Some researchers have found that low-income students are more likely to be bullied (Hong & Espelage, 2012), and boys and girls whose parents are unemployed are more likely to experience sexual harassment (Kaltiala-Heino et al., 2016; Tillyer et al., 2010). Relatedly, Tillyer et al. (2010) found that among girls, but not boys, a lower parental educational level was significantly associated with higher SH victimization. Finally, Kaltiala-Heino et al. (2016) found that living in a single parent home was associated with experiencing higher levels of sexual harassment across three specific forms—disturbing sexual propositions, sexual name-calling, and unwanted sexual touching.

In terms of sexual harassment, some researchers have emphasized the impactful role that parents play in gender socialization, often in alignment with broader cultural norms around dating and heteronormative gender roles (Brown et al., 2020). For example, parents often reward boys for aggressive behavior and reward girls for passive behaviors and alignment with broader gender roles, which can contribute to later involvement in sexual harassment.

This is one of the few levels where researchers have identified protective factors against bullying and sexual harassment. For example, positive parenting behaviors including parental support, parental involvement, open and honest communication, and affectionate parent–youth relationships serve as protective factors against bullying (Lereya et al., 2013). Doty et al. (2017) found that students who had more positive communication with their parents and who had parents and teachers who cared about them were less likely to experience/perpetrate bullying and sexual harassment (Doty et al., 2017). Kaltiala-Heino et al. (2016) found that parental involvement in the adolescent’s personal life is associated with a lower likelihood of experiencing sexual harassment, hence it may serve as a protective factor for sexual harassment.

The School Survey on Crime and Safety administered by the U.S. Department of Education includes a question about parental involvement, suggesting that parents are an important stakeholder group. The survey identifies formal processes inviting parental input on policies related to school safety and providing assistance to parents in addressing student problematic behavior as potential forms of parental involvement (Kaatz et al., 2024). Schools vary in their parental involvement, given that 56.41% of schools had a formal process to gather parental input on policies and 49.75% offered assistance or training to parents in addressing student problematic behavior (Kaatz et al., 2024).

#### 4.2.2. Peers

Bullying literature shows that peer relationships can be a protective factor as well as a risk factor for bullying due to the characteristics of friends. A systematic review by Ttofi et al. (2014) finds that prosocial peers are important protective factors against bullying perpetration and bullying victimization. High-quality support in friendships is also demonstrated as an important protective factor against bullying perpetration (Kendrick et al., 2012; Bollmer et al. 2005) and bullying victimization (Kendrick et al., 2012). Johns et al. (2020) suggests that school organizations such as gay–straight alliances can play a positive role in improving school climate for LGB students.

Nonetheless, there are other studies in the literature that point to peer interactions as a potential risk factor for bullying perpetration and victimization. Students who belong to the same friendship group are found to be comparable in terms of their direct involvement and support for bullying behavior, and students who belong to friendship groups with a norm of bullying are more likely to engage in bullying perpetration than members of groups without such a norm (Duffy & Nesdale, 2008). Interaction with antisocial friends is also found to be a risk factor for bullying perpetration (Hemphill et al., 2014). In contrast, peer rejection and social isolation are found to be risk factors for bullying victimization (Cook et al., 2010; Kljakovic & Hunt, 2016).

Some researchers note the important role that peers play in gender socialization, often encouraging and rewarding adherence to traditional gender roles and norms, such as encouraging aggression among boys and sexual objectification of girls (Brown et al., 2020; Holmqvist Gattario & Lunde, 2023). These processes can contribute to gender/sexual harassment of boys who are not viewed as strong or aggressive and to the sexual harassment and objectification of girls. “Much of the teasing and bullying that is directed to gender-atypical (i.e., non-hegemonic) boys centers on their (presumed) sexual orientation, most often including homophobic slurs and comments” (Brown et al., 2020, p. 307).

It is also important to understand the relationships between perpetrators and victims when assessing these forms of violence, but not all studies collect those data. Basile et al. (2020) point out that, consistent with the literature, half of the students in their study who reported sexual violence victimization were victimized by someone other than a dating partner. The authors suggest that, “Indeed, perpetrators of sexual violence during youth can be acquaintances, family members, persons in a position of authority, and strangers, in addition to dating partners” (Basile et al., 2020, p. 5). Brown et al. (2020) further argue that sexual harassment usually takes place in public spaces in schools and is witnessed, and often encouraged, by peers, leading to the normalization of such behaviors.

#### 4.2.3. Teachers and Other Staff

Relationships with teachers and adults can be an important protective factor against bullying and sexual harassment perpetration and victimization. Students who report higher teacher support and who perceive their teachers as fair, supportive, and interested in them are less likely to bully (Barboza et al., 2009). Students who have teachers who care about them are less likely to become victims or perpetrators of bullying and sexual harassment (Doty et al., 2017).

Similarly, bullying is less likely to occur in schools when teachers respond quickly and effectively to bullying incidents and school staff members have attitudes not supportive of bullying incidents (Olweus, 1993). The frequency of bullying perpetration is higher for students who have thought their teachers would not penalize them compared to those students who have thought their teachers would penalize them (DeSouza & Ribeiro, 2005).

On the other hand, researchers suggest that teachers’ and staff members’ tolerance of sexual harassment serves as a risk factor and can contribute to its acceptance and normalization (Horn & Poteat, 2023; Ormerod et al., 2023). Several studies have demonstrated that teachers do not understand sexual harassment, have not received sufficient training, or do not know how to intervene (Edwards et al., 2020; Charmaraman et al., 2013). Some are only aware of sexual harassment occurring between adults (Brown et al., 2020; Edwards et al., 2020) or adult-to-student situations, making them not prepared to address sexual harassment among students (Charmaraman et al., 2013). Staff and teachers are often dismissive of sexual harassment (Espelage et al., 2016; Rolfe & Schroeder, 2020). Research suggests, however, that students are more likely to believe that sexual harassment is wrong when teachers intervene (Skoog et al., 2023) and students are less likely to perpetuate sexual harassment if they believe that it is wrong and harmful (Peter et al., 2016). In addition, young women who perceived their school as failing to respond to sexual harassment experienced more frequent sexual harassment and felt less safe at school (Ormerod et al., 2023).

Although there is a growing literature on the role of school resource officers in addressing school safety issues, including the Government Accountability Office’s annual report, little attention has been given to their role in addressing school bullying or sexual harassment. Some research does suggest that the presence of school resource officers has contributed to increased arrests of students, but it is not clear whether bullying or sexual harassment behaviors are responded to in this way.

### 4.3. Mesosystem

In this section, we examine school policies, practice, prevention efforts, and staff training related to bullying and sexual harassment. These risk and protective factors can contribute to specific contexts and climates that have an impact on student behaviors and feelings of belonging and safety.

#### 4.3.1. School Climate

Ormerod et al. (2023) suggest that school climate is a complex and multifaceted construct that is difficult to define. “School climate is based on patterns of people’s experiences of school life and reflects norms, goals, values, interpersonal relationships, teaching and learning practices, and organizational structures” (Cohen et al., 2009, p. 180). School climate can be defined and operationalized in terms of the school environment and individual perceptions and feelings of belonging and can include both organizational and social dimensions. For example, studies suggest that low school connectedness (such as a low sense of belonging in school and less positive school climate perceptions) and negative school environment (such as lower levels of adult supervision, attitudes tolerant of bullying behaviors, and unpleasant, unfair, and unwelcoming school atmosphere) are risk factors for bullying perpetration and victimization (Hong & Espelage, 2012; Barboza et al., 2009; O’Brien, 2021; Cook et al., 2010). Similarly, higher levels of classroom status hierarchy, and injunctive norms on bullying (e.g., less negative attitudes towards bullying and acceptance of aggressive behavior among students) (Pouwels & Garandeau, 2021) have been associated with increased risk of bullying.

Ormerod et al. (2023) found that school climates that are tolerant of sexual harassment are important risk factors and contribute indirectly to student disengagement from school through peer sexual harassment. Brown et al. (2020) similarly suggest that most sex education classes do not address sexual harassment, missing an opportunity to enhance protective factors through providing accurate information and communication skills.

#### 4.3.2. Structural Features of the School Environment

Studies show mixed findings concerning the impacts of classroom size on bullying. Even though few studies find that bullying is less likely to occur in smaller classrooms due to a higher adult/child ratio, most studies have found either no association or negative association between bullying and classroom size (Pouwels & Garandeau, 2021). The higher prevalence of bullying in smaller classrooms can be explained by a relatively higher power imbalance between perpetrators and victims in smaller classrooms as it is easier for perpetrators to gain social power while it is more difficult for victims to find peer support when the number of classmates is very small (Pouwels & Garandeau, 2021).

Espelage et al. (2016) also suggest that researchers in psychology, criminology, and urban planning have examined where bullying, dating violence, and sexual harassment occur at school through “hot spot” analyses, but sexual harassment has received the least attention and findings are mixed. Most of these studies identify hallways and classrooms as the primary spaces in which sexual harassment occurs (Brown et al., 2023; Espelage et al., 2016; Interactive et al., 2005). In general, sexual harassment takes place in environments with low adult supervision (Skoog et al., 2023; Rolfe & Schroeder, 2020).

#### 4.3.3. School Prevention Policies & Staff Training

A difference between bullying and sexual harassment is that there is no federal law that addresses bullying at schools; instead bullying is regulated by state laws and individual school district policies (Gruber & Fineran, 2016). Sexual harassment, on the other hand, is prohibited at the federal level through Title IX, which is monitored by the U.S. Department of Education Office of Civil Rights (Brown et al., 2023). “While Title IX does not ‘naturally’ cover bullying under ‘sexual harassment language’ the court has declared that when bullying is based on sex or gender and is pervasive or severe, it can be a violation of Title IX” (Miller & Mondschein, 2017, p. 192).

Anti-bullying laws differ by the state in terms of their bullying definitions, reporting and investigation procedures, and prevention and training provisions (Cascardi et al., 2018). Cascardi et al. (2018), for example, found that 84% of states required training for school staff while only 4% required bystander education, 16% harassment education–sensitivity training, and 2% healthy relationships education. Around half of the anti-bullying laws include an enumeration of student groups who are explicitly protected under school district bullying and harassment policies (Cascardi et al., 2018). The most common protected student groups included in bullying and harassment policies are race, national origin, religion, disability, and sex, while some policies also include sexual orientation, gender identity, age, physical appearance, and socioeconomic status as protected characteristics (Stuart-Cassel et al., 2011).

Researchers find varying degrees of compliance with Title IX within schools (Brown et al., 2023). Brown and colleagues, for example, found that “only one-fourth of the school districts had SH policies in which SH was clearly defined and in which there were noted consequences for perpetrating SH” (Brown et al., 2023, p. 1411). In addition, studies show a lack of awareness of these policies by students and teachers alike (Brown et al., 2023). For example, Brown et al. (2023) found that students were more aware of bullying policies than sexual harassment policies. Additionally, Charmaraman et al. (2013) found that teachers received more training about bullying than sexual harassment and they were more interested in receiving additional training about bullying and district bullying policies than improving their understanding about sexual harassment at schools.

Researchers also suggest the importance of investigating both the objective aspects of the policies’ structural elements, as well as students’ perceptions of the climate, to their understanding of the impact of policies. The effects of school-based programs to reduce school violence have been researched by a number of scholars reporting consistent findings, with some variations in overall findings associated with methodological differences. In general, comprehensive systematic reviews and meta-analyses have indicated that anti-bullying school programs are effective in reducing bullying perpetration and bullying victimization (Ttofi & Farrington, 2011; Jiménez-Barbero et al., 2016; Gaffney et al., 2019). Russell et al. (2010), for example, suggest that supportive school policies such as non-discrimination and anti-bullying policies enumerating or including sexual orientation or gender identity is important for safety and wellbeing of LGBTQ youth in schools. Furthermore, it is found that teachers are more likely to intervene in sexual harassment if schools have explicit anti-sexual harassment policies and support in implementing these policies (Doty et al., 2017). In addition to policies, studies in the literature indicate that parent training/meetings, improved playground supervision, disciplinary methods, classroom management, and teacher training are identified as the most important program elements that are associated with a decrease in bullying (Ttofi & Farrington, 2011; Waasdorp et al., 2021).

The School Survey on Crime and Safety administered by the U.S. Department of Education includes a list of programs schools might employ to address various forms of violence. For example, the survey of the 2019–2020 academic year found that 95.19% of schools had specific prevention curricula focused on forms of violence, 92.91% of schools offered social and emotional learning programs, 10.34% utilized student courts, 58.73% employed restorative practices, and 87.51% offered “programs to promote a sense of community or social integration among students” (Kaatz et al., 2024). There is a dearth of research evaluating the effectiveness of these types of programs, especially in the United States.

### 4.4. Exosystem

In this section, we address the influence of neighborhoods and school boards on bullying and sexual harassment in schools. Even though structural features of the school environment (class size, composition of the school peer groups, etc.) have been examined in the literature, relatively little empirical research has focused on the immediate social environments of the community and neighborhood (Schwartz et al., 2021).

#### 4.4.1. Neighborhoods and Communities

Neighborhoods may present risk factors that increase the likelihood of bullying perpetration and victimization due to inadequate adult oversight, lack of age segregation that minimizes children’s contact with older and larger adolescents, or negative peer influences (Schwartz et al., 2021). Existing studies (e.g., Chaux et al., 2009; Davis et al. 2020) suggest that unsafe neighborhood environments and exposure to neighborhood violence are risk factors for bullying behavior. Research finds that witnessing community violence increases bullying behavior or other forms of aggression by influencing children to imitate violent acts and develop social cognitions (Guerra et al., 2003; Schwartz & Proctor, 2000).

Neighborhood economic disadvantage and low levels of social capital and social cohesion in the neighborhood are also found to be risk factors for bullying. Compared to their peers in affluent communities, children residing in disadvantaged neighborhoods (neighborhoods with a higher proportion of households receiving public assistance, a higher proportion of not married families, a higher number of official bullying incidents, and lower levels of collective efficacy) are more likely to become the targets or perpetrators of bullying (Han et al., 2019). According to Bowes et al. (2009), young children living in areas with high levels of neighborhood conflicts (arguments among neighbors) were more likely to become bully victims at a later time point.

Very little research has focused on protective factors for school-based violence at this social-ecological level. One exception is Johns et al. (2020), who suggest that schools can partner with local organizations to provide support for marginalized students. They provide a link to the Center for Disease Control’s listing of such prevention programs (https://www.cdc.gov/violence-prevention/php/funded-programs-opportunities/index.html, accessed on 11 January 2025).

#### 4.4.2. School Boards

School boards often have decision-making power over school policies and programming that can influence bullying and sexual harassment, creating risk or protective factors. Some, for example, have opposed teaching sex education and critical race theory, as well as social emotional learning initiatives, all of which have been shown to help reduce bullying and sexual harassment (Abrams, 2023). In addition, many school boards are currently heavily engaged in promoting or opposing policies regarding transgender students’ engagement in sports (Aclu Virginia, 2024). Such efforts prevent schools from creating protective factors including more respectful and inclusive climates and can work to exacerbate disrespect and hatred of marginalized groups.

The School Survey on Crime and Safety also recognizes the potential for community groups to be involved in school safety, asking about the involvement of various groups in promoting school safety. Such groups include parent groups, social service agencies, juvenile justice agencies, law enforcement agencies, mental health agencies, civic organizations/service clubs, private corporations or businesses, and religious organizations (Kaatz et al., 2024).

### 4.5. Macrosystem

In this section, we address historical and broader social and cultural influences on bullying and sexual harassment. Research suggests that although they are external to schools, these factors can contribute to students’ views of particular social groups and normalization of hate and violence.

#### 4.5.1. Broader Culture, Social and Political Climate

The body of research on social and cultural factors associated with both forms of violence is limited (Basile et al., 2009), but studies suggest that these factors can create important risk or protective factors. Williams and Guerra (2007), for example, found that normative beliefs approving of bullying increase the likelihood of bullying. Some studies suggest a link between homophobic bullying and traditional masculinity, heteronormative culture, and heterosexist language (e.g., Phoenix et al., 2003; Hong & Garbarino, 2012).

Studies have documented that political events such as the presidential election of Donald Trump significantly affected bullying and particularly, bias-based bullying behaviors by shaping cultural norms and increasing violent and divisive language in the media (Gill & Govier, 2023; Huang & Cornell, 2019). Huang and Cornell (2019) found that there were higher rates of bullying incidents in counties supporting the Republican candidate after the 2016 U.S. presidential election even though there were no significant differences in bullying incidents between Democratic and Republican favoring counties before the election.

Gruber and Fineran (2008) suggest that “sexual harassment is more directly and clearly related to hegemonic masculinity [than is bullying] and therefore taps into potent structural and culturally-sanctioned roles and meanings (masculine–feminine, heterosexual–homosexual) that are central components of social stratification” (Gruber & Fineran, 2008, p. 2). Similarly, Brown et al. (2020) suggest that “the sexualization of women and girls and hegemonic masculinity among men and boys work together to create a script for how boys and girls, and men and women, should interact with one another” (Brown et al., 2020, p. 301). This script socializes boys to be more sexually aggressive and to associate sexual conquests and promiscuity with masculinity and socializes girls to be passive and seek boys’ desire.

Herrera Hernandez and Oswald (2023) studied young people’s attitudes towards the confirmation of U.S. Supreme Court Justice Brett Kavanaugh in 2018 and their attitudes toward sexual harassment. Judge Kavanaugh had been accused of sexual assault. The authors found that students who were more accepting toward the confirmation of Judge Kavanaugh had more accepting attitudes toward sexual harassment.

#### 4.5.2. Media

Media is a common method of disseminating cultural norms, and young people are increasingly consuming more kinds of information from a variety of forms and outlets. Much of the research in this area has identified risk, rather than protective, factors. For instance, research consistently finds that exposure to high television viewing (Kuntsche, 2004; Zimmerman et al., 2005; Barboza et al., 2009) increases the likelihood of bullying others. Also, children, particularly boys, who are exposed to excessive video game playing (Olson et al., 2009; Yang, 2012; Kuntsche, 2004) are more likely to bully or cyberbully others. Furthermore, a review of the literature by Fanti and Zacharaki (2021) indicates that exposure to violence in the media (such as violent television programs, movies, video games, and violent scenes on the internet) is an important risk factor for bullying and cyberbullying behavior.

Brown et al. (2020) suggest that a variety of media, as well as children’s toys, contribute to the normative gender socialization patterns that contribute to sexual harassment. Movies, television, and online pornography, for example, all sexually objectify girls and valorize masculine sexual aggression. Harris and Kruger (2023) also describe how hip hop culture portrays Black girls as hypersexual, which can contribute to their experiences of sexual harassment.

## 5. Discussion

Our review of similarities and differences between school-based bullying and sexual harassment literature focused on students’ experiences of these forms of violence, and the risk and protective factors provided by stakeholders across the social-ecological spectrum. Consistent with other researchers (Cook et al., 2010; Gruber & Fineran, 2008) we found that a greater number of studies of both forms of violence focus on student and microsystem-level factors rather than on higher levels of the ecosystem. In addition, the research overwhelmingly identifies more risk factors than protective factors. Finally, we found more similarities than differences between the two forms of school-based violence.

### 5.1. Student-Level

Although there are mixed findings in terms of demographic characteristics as risk factors for harassing behaviors, research generally suggests that members of marginalized groups, i.e., those with less power and status, are at risk for victimization of bullying and sexual harassment. Girls and transgender/nonbinary students, members of racial/ethnic and religious minority groups, LGB students, and students with disabilities experience disproportionate levels of these behaviors. Although many researchers have focused on individual-level factors, fewer have investigated the intersections of multiple identities and various experiences of bullying and sexual harassment including polyvictimization. For example, we know little about how gender, race/ethnicity, and disability intersect to create risk and protective factors; we lack understanding of variations within single identity groups. Research often fails to identify the identities of the both perpetrator and victim, and particularly in the bullying literature, the relationships between perpetrator and victim, limiting our understanding of how these factors combine to create risk and protective factors.

### 5.2. Microsystem

Existing research suggests that various stakeholders can play an important role in encouraging or preventing both types of harassing behaviors in schools, and each group should be engaged in prevention and intervention efforts. For example, parental attitudes and behaviors regarding gender roles and toward members of specific identity groups can either promote or oppose equality and respectful treatment. Other family factors, e.g., employment, socioeconomic status, family structure, and educational level have been shown to be associated with bullying and sexual harassment, suggesting that family stability and security serve as protective factors.

Peers have tremendous influence during adolescence, and the status hierarchies embedded within school settings create opportunities for pressure to engage in harassing behaviors. Young people are developing sexual identities and experiencing desire, and those with less social power and status are at greater risk for both types of abuse. Such abuse reinforces norms and hierarchies. Young people are exploring new social roles and their pursuit of status among peers in their new school settings should be examined more with respect to harassing behaviors and in terms of potential interventions. For example, interventions could include effective school transition programs for students entering middle or high school and could focus on leadership development that emphasizes respect and greater inclusion as a protective factor.

Several studies suggest the important role of teachers in setting the tone regarding harassing behaviors. When teachers support and promote anti-harassment policies and intervene when behaviors occur, students experience a more positive school climate and are more engaged academically, creating strong protective factors.

### 5.3. Mesosystem, Exosystem, and Macrosystem

These higher levels of the social-ecological model receive much less attention from researchers, but research suggests that policies and school climate at the mesosystem, and broad cultural norms at the macro level influence students’ attitudes and behaviors. Much of the research on policies is limited to the mere existence of policies with little investigation into policy implementation and effectiveness. Some research suggests that teacher, staff, and student awareness of and support for those policies as well as the involvement of the school community in the policy development are just as important as the existence of the policies, related to the “shared school vision” construct of the model.

Very little attention has been given to the risk and protective factors for either behavior at the exosystem level. Neighborhood influence can be conceptualized as socioeconomic, as well as political factors, both of which have been shown to influence harassment. These factors overlap with the macro level when they lead to policies and legislation. Government-driven educational reform, legislation, and data collection are identified as important macro-level influences on school-based violence, but we found limited research on these broader reforms. These are related to, and informed by, school board and lawmaker decisions, and legislation is a dynamic area of influence on harassing behaviors. Changes to legal protections for marginalized groups have occurred at the state and federal levels in recent years, shaping schools’ prevention of and response to bullying and sexual harassment.

Cultural norms appear to influence both behaviors as well, including specific contexts and content of messages regarding gender roles and expectations that target marginalized groups including students of color, girls, and LGBTQA+ students. Simply identifying the identities that are at greater risk for experiencing harassing behaviors is insufficient to promote change, however. Making the connections between the individual-level risk and the social and cultural levels of influence through, for example, the media, school boards, and legislation, is key.

## 6. Conclusions, Limitations, and Future Directions

Our review identifies risk and protective factors across two prevalent forms of school-based violence at each of the social-ecological levels. Emphasizing similarities and differences at each level, as well as connections across the levels, provides new insight into these forms of violence and suggests opportunities for future research, theories, and policies.

Our review makes two primary contributions to the existing literature examining risk and protective factors of school-based bullying and sexual harassment. First, while there are some reviews in the bullying literature that used the social-ecological model, studies have not applied this model to the school setting or educational context. Consequently, they have not included many of the factors we included based on Allen et al.’ (2018) school-specific model such as school prevention policies, school board, school vision, and government reforms. Secondly, no existing study compares risk and protective factors of bullying and sexual harassment across the social-ecological model.

Regarding the limitations of the current review, it is important to highlight that the authors have conducted a narrative review. Due to the nature of the narrative review, there might be bias in the selection of articles for review. Even though the narrative review methodology allowed us to reformulate our search terms throughout the literature search process and resulted in a wider review encompassing the role of all important stakeholders in the school community, we believe that systematic reviews might add to this discussion. Further research might follow acknowledged guidelines for a review like PRISMA. Further research adopting a systematic review methodology can also include search terms like “cultural norms”, and “school prevention policies” along with bullying and/or sexual harassment to identify more studies at the higher levels of the social-ecological model. It is also important to specify the school context (like K-12 and secondary schools) while writing queries, particularly for sexual harassment, as these behaviors occur throughout the society. Also, narrative reviews or systematic reviews on this topic might be restricted to a particular geographic area due to different cultural definitions of bullying and sexual harassment.

In terms of research, our review suggests that we need more consistency across studies regarding definitions and measures of various forms of school based violence. On the one hand, we need to use more consistent definitions of forms of violence to ensure that researchers are measuring the same thing and allow for comparability. On the other hand, we need to include variations in experiences that occur across distinctive groups of students in order to capture specific risk and protective factors as well as outcomes. For example, we need to distinguish between cyberbullying and online sexual harassment (Copp et al., 2021) and sexual violence, sexual harassment, and dating violence. We also need to distinguish between sexual harassment, sexual bullying, and gender-based bullying as these terms may be used interchangeably. We need to pay more attention to the victim/perpetrator/witness overlap (Valik et al., 2023) and clearly measure each experience. This includes identifying the identities of each role and assessing patterns in both victimization and perpetration. We also need to incorporate more intersectional approaches that examine students’ unique experiences across and within gender, race/ethnicity, sexual orientation, and disability.

Research should focus on the higher levels of the social-ecological model to better understand how school climate, neighborhoods, and broader cultural messages influence bullying and sexual harassment. The increasing power of school boards to shape policies related to sports participation, bathroom accessibility, and teaching critical race theory and sex education that affect respect for, and acceptance of, marginalized students, is another topic that requires further study.

It is important to develop theories that help to explain both similarities and differences in factors that influence both types of behaviors. Although the content of the motivation, e.g., misogyny, racism, homophobia, transphobia, xenophobia, or ableism, might vary, many of the dynamics, tactics, and consequences of the two types of harassment operate in similar ways. Theories should address intersecting identities as well as connections across the levels of the social-ecological model.

The findings suggest implications for policy and practice as well. Policies should address the various forms of bullying and sexual harassment that students experience and what is unique about each. Given the differences in prevalence rates of each type of violence by gender, race/ethnicity, religion, sexual identity, and disability, prevention programs should be developed that address the unique needs of each of these groups. School-based interventions that address bias and prejudice in peer victimizations are very limited (Earnshaw et al., 2018). Earnshaw et al. (2018), for example, suggest adopting multicomponent interventions that address distinct bias-related factors such as changes to attitudes and knowledge concerning social dominance orientation, stereotypes, and prejudice. Also, not all students experience these two forms of violence in the same ways, as perpetrators or victims. Even though similar broad factors may contribute to the violence, students’ engagement in particular tactics and their experiences of consequences can be unique. Research identifying protective factors at each level could contribute to more effective prevention programs.

Bullying and sexual harassment involve more individuals than the victim and perpetrator. Stakeholders, including teachers, parents, school staff, peers, and the broader community, all contribute to these behaviors through failing to respond, encouraging violent behaviors, creating violence-supportive norms, cultures, and environments. Our review finds that each social-ecological level includes risk factors that increase the likelihood of bullying and sexual harassment perpetration and victimization. Moreover, these harassing behaviors and hostile environments not only affect the student victims but also the larger school community. Hence, we support an ecological approach to the prevention of bullying and sexual harassment.

Interventions and supportive responses should be made available to students at risk for both victimization and perpetration at all levels of the ecological system. The policy regarding sexual harassment recognizes behaviors that create a “hostile environment” for students (Brown et al., 2023). Hence, schools may be required “to address the school norms and climate that allow for (or do not discourage) harassing actions, such as unwanted sexual remarks or contact” (Cascardi et al., 2018, p. 3286). However, schools are not obliged to address the hostile environment if they identify the behavior as bullying and interventions to bullying may be limited to disciplinary consequences for the aggressor and individual-level referrals or counseling for the victim (Cascardi et al., 2018, p. 3286). Such responses fail to address the broader influences such as hostile school climates. Interventions such as social emotional learning programs, bystander programs, changes to the built environment, and school connectedness programs address these higher levels of influence. Finally, our review suggests that some policies and interventions do address the mesosystem, exosystem, and macro levels, but they must be known, implemented, and supported by all stakeholders to be effective. Coordinated efforts across the entire ecological system, including families, schools, and communities, are required to address bullying and sexual harassment effectively.

Overall, preventing any form of harassment at school will benefit not only students but also the entire school community (Miller & Mondschein, 2017). The causes of school-based harassment are complex, and solving these problems will require stronger theories, better research, and comprehensive prevention, intervention, and response approaches. Ultimately, all community stakeholders must be engaged in all of these endeavors for them to be successful.

## References

[B1-behavsci-15-00061] Abrams Z. (2023). Teaching social-emotional learning is under attack. Monitor on Psychology.

[B2-behavsci-15-00061] Aclu Virginia (2024). Update on the status of the 2023 VDOE model policies.

[B3-behavsci-15-00061] Allen K., Kern M. L., Vella-Brodrick D., Hattie J., Waters L. (2018). What schools need to know about fostering school belonging: A meta-analysis. Educational Psychology Review.

[B4-behavsci-15-00061] Álvarez-García D., García T., Núñez J. C. (2015). Predictors of school bullying perpetration in adolescence: A systematic review. Aggression and Violent Behavior.

[B5-behavsci-15-00061] Baldry A. C. (2003). Bullying in school and exposure to domestic violence. Child Abuse & Neglect.

[B6-behavsci-15-00061] Barboza G. E., Schiamberg L. B., Oehmke J., Korzeniewski S. J., Post L. A., Heraux C. G. (2009). Individual characteristics and the multiple contexts of adolescent bullying: An ecological perspective. Journal of Youth and Adolescence.

[B7-behavsci-15-00061] Basile K. C., Clayton H. B., DeGue S., Gilford J. W., Vagi K. J., Suarez N. A., Zwald M. L., Lowry R. (2020). Interpersonal violence victimization among high school students—Youth risk behavior survey, United States, 2019. MMWR Supplements.

[B8-behavsci-15-00061] Basile K. C., Espelage D. L., Rivers I., McMahon P. M., Simon T. R. (2009). The theoretical and empirical links between bullying behavior and male sexual violence perpetration. Aggression and Violent Behavior.

[B9-behavsci-15-00061] Bauer N. S., Herrenkohl T. I., Lozano P., Rivara F. P., Hill K. G., Hawkins J. D. (2006). Childhood bullying involvement and exposure to intimate partner violence. Pediatrics.

[B10-behavsci-15-00061] Blake J. J., Lund E. M., Zhou Q., Kwok O. M., Benz M. R. (2012). National prevalence rates of bully victimization among students with disabilities in the United States. School Psychology Quarterly.

[B11-behavsci-15-00061] Bollmer J. M., Milich R., Harris M. J., Maras M. A. (2005). A friend in need: The role of friendship quality as a protective factor in peer victimization and bullying. Journal of Interpersonal Violence.

[B12-behavsci-15-00061] Bowes L., Arseneault L., Maughan B., Taylor A., Caspi A., Moffitt T. E. (2009). School, neighborhood, and family factors are associated with children’s bullying involvement: A nationally representative longitudinal study. Journal of the American Academy of Child & Adolescent Psychiatry.

[B13-behavsci-15-00061] Bronfenbrenner U. (1979). Ecology of human development: Experiments by nature and design.

[B14-behavsci-15-00061] Brown C. S., Biefeld S. D., Bulin J. (2023). High school policies about sexual harassment: What’s on the books and what students think. Journal of Social Issues.

[B15-behavsci-15-00061] Brown C. S., Biefeld S. D., Elpers N. (2020). A bioecological theory of sexual harassment of girls: Research synthesis and proposed model. Review of General Psychology.

[B16-behavsci-15-00061] Brubaker S. J. (2019). Theorizing gender violence.

[B17-behavsci-15-00061] Byrne J. A. (2016). Improving the peer review of narrative literature reviews. Research Integrity and Peer Review.

[B18-behavsci-15-00061] Cascardi M., King C. M., Rector D., DelPozzo J. (2018). School-based bullying and teen dating violence prevention laws: Overlapping or distinct?. Journal of Interpersonal Violence.

[B19-behavsci-15-00061] Charmaraman L., Jones A. E., Stein N., Espelage D. L. (2013). Is it bullying or sexual harassment? Knowledge, attitudes, and professional development experiences of middle school staff. Journal of School Health.

[B20-behavsci-15-00061] Chaux E., Molano A., Podlesky P. (2009). Socio-economic, socio-political and socio emotional variables explaining school bullying: A country-wide multilevel analysis. Aggressive Behavior.

[B21-behavsci-15-00061] Clear E. R., Coker A. L., Cook-Craig P. G., Bush H. M., Garcia L. S., Williams C. M., Lewis A. M., Fisher B. S. (2014). Sexual harassment victimization and perpetration among high school students. Violence Against Women.

[B22-behavsci-15-00061] Cohen J., Mccabe E. M., Michelli N. M., Pickeral T. (2009). School climate: Research, policy, practice, and teacher education. Teachers College Record.

[B23-behavsci-15-00061] Collins J. A., Fauser B. C. (2005). Balancing the strengths of systematic and narrative reviews. Human Reproduction Update.

[B24-behavsci-15-00061] Cook C. R., Williams K. R., Guerra N. G., Kim T. E., Sadek S. (2010). Predictors of bullying and victimization in childhood and adolescence: A meta-analytic investigation. School Psychology Quarterly.

[B25-behavsci-15-00061] Copp J. E., Mumford E. A., Taylor B. G. (2021). Online sexual harassment and cyberbullying in a nationally representative sample of teens: Prevalence, predictors, and consequences. Journal of Adolescence.

[B26-behavsci-15-00061] Craig W. M., Pepler D., Connolly J., Henderson K., Juvonen J., Graham S. (2001). Developmental context of peer harassment in early adolescence. Peer harassment in school: The plight of the vulnerable and victimized.

[B27-behavsci-15-00061] Cutbush S., Williams J., Miller S. (2016). Teen dating violence, sexual harassment, and bullying among middle school students: Examining mediation and moderated mediation by gender. Prevention Science.

[B28-behavsci-15-00061] Davis J. P., Ingram K. M., Merrin G. J., Espelage D. L. (2020). Exposure to parental and community violence and the relationship to bullying perpetration and victimization among early adolescents: A parallel process growth mixture latent transition analysis. Scandinavian Journal of Psychology.

[B29-behavsci-15-00061] DeSouza E. R., Ribeiro J. A. (2005). Bullying and sexual harassment among Brazilian high school students. Journal of Interpersonal Violence.

[B30-behavsci-15-00061] Doty J. L., Gower A. L., Rudi J. H., McMorris B. J., Borowsky I. W. (2017). Patterns of bullying and sexual harassment: Connections with parents and teachers as direct protective factors. Journal of Youth and Adolescence.

[B31-behavsci-15-00061] Duffy A. L., Nesdale D. (2008). Peer groups, social identity, and children’s bullying behavior. Social Development.

[B32-behavsci-15-00061] Earnshaw V. A., Reisner S. L., Menino D. D., Poteat V. P., Bogart L. M., Barnes T. N., Schuster M. A. (2018). Stigma-based bullying interventions: A systematic review. Developmental Review.

[B33-behavsci-15-00061] Edwards K. M., Rodenhizer K. A., Eckstein R. P. (2020). School personnel’s bystander action in situations of dating violence, sexual violence, and sexual harassment among high school teens: A qualitative analysis. Journal of Interpersonal Violence.

[B34-behavsci-15-00061] Ellis W. E., Wolfe D. A. (2015). Bullying predicts reported dating violence and observed qualities in adolescent dating relationships. Journal of Interpersonal Violence.

[B35-behavsci-15-00061] Espelage D. L., Basile K. C., De La Rue L., Hamburger M. E. (2015). Longitudinal associations among bullying, homophobic teasing, and sexual violence perpetration among middle school students. Journal of Interpersonal Violence.

[B36-behavsci-15-00061] Espelage D. L., Hong J. S., Rinehart S., Doshi N. (2016). Understanding types, locations, & perpetrators of peer-to-peer sexual harassment in US middle schools: A focus on sex, racial, and grade differences. Children and Youth Services Review.

[B37-behavsci-15-00061] Fanti K. A., Zacharaki G., Smith P. K., Norman J. O. H. (2021). Media factors and bullying. The Wiley Blackwell handbook of bullying: A comprehensive and international review of research and intervention.

[B38-behavsci-15-00061] Ferrari R. (2015). Writing narrative style literature reviews. Medical Writing.

[B39-behavsci-15-00061] Fineran S., Bolen R. M. (2006). Risk factors for peer sexual harassment in schools. Journal of Interpersonal Violence.

[B40-behavsci-15-00061] Foshee V. A., McNaughton Reyes H. L., Chen M. S., Ennett S. T., Basile K. C., DeGue S., Vivolo-Kantor A. M., Moracco K. E., Bowling J. M. (2016). Shared risk factors for the perpetration of physical dating violence, bullying, and sexual harassment among adolescents exposed to domestic violence. Journal of Youth and Adolescence.

[B41-behavsci-15-00061] Foshee V. A., McNaughton Reyes H. L., Vivolo-Kantor A. M., Basile K. C., Chang L. Y., Faris R., Ennett S. T. (2014). Bullying as a longitudinal predictor of adolescent dating violence. Journal of Adolescent Health.

[B42-behavsci-15-00061] Gaffney H., Ttofi M. M., Farrington D. P. (2019). Evaluating the effectiveness of school-bullying prevention programs: An updated meta-analytical review. Aggression and Violent Behavior.

[B43-behavsci-15-00061] Gill M., Govier D. (2023). Bias-based bullying in California schools: The impact of the 2016 election cycle. Journal of Interpersonal Violence.

[B44-behavsci-15-00061] Gladden R. M., Vivolo-Kantor A. M., Hamburger M. E., Lumpkin C. D. (2014). Bullying surveillance among youths: Uniform definitions for public health and recommended data elements, version 1.0.

[B45-behavsci-15-00061] Goli S., Rahimi F., Goli M. (2022). Experiences of teachers, educators, and school counselors about the sexual and reproductive health of educable intellectually disabled adolescent girls: A qualitative study. Reproductive Health.

[B46-behavsci-15-00061] Government Accountability Office (2021). Students’ experiences with bullying, hate speech, hate crimes, and victimization in schools.

[B47-behavsci-15-00061] Green B. N., Johnson C. D., Adams A. (2006). Writing narrative literature reviews for peer-reviewed journals: Secrets of the trade. Journal of Chiropractic Medicine.

[B48-behavsci-15-00061] Gruber J. E., Fineran S. (2008). Comparing the impact of bullying and sexual harassment victimization on the mental and physical health of adolescents. Sex Roles.

[B49-behavsci-15-00061] Gruber J., Fineran S. (2016). Sexual harassment, bullying, and school outcomes for high school girls and boys. Violence Against Women.

[B50-behavsci-15-00061] Grych J., Swan S. (2012). Toward a more comprehensive understanding of interpersonal violence: Introduction to the special issue on interconnections among different types of violence. Psychology of Violence.

[B51-behavsci-15-00061] Guerra N. G., Huesmann L. R., Spindler A. (2003). Community violence exposure, social cognition, and aggression among urban elementary school children. Child Development.

[B52-behavsci-15-00061] Han Y., Kim H., Ma J., Song J., Hong H. (2019). Neighborhood predictors of bullying perpetration and victimization trajectories among South Korean adolescents. Journal of Community Psychology.

[B53-behavsci-15-00061] Harris J., Kruger A. C. (2023). “We always tell them, but they don’t do anything about it!” Middle school Black girls experiences with sexual harassment at an urban middle school. Urban Education.

[B54-behavsci-15-00061] Hemphill S. A., Tollit M., Herrenkohl T. I. (2014). Protective factors against the impact of school bullying perpetration and victimization on young adult externalizing and internalizing problems. Journal of School Violence.

[B55-behavsci-15-00061] Herrera Hernandez E., Oswald D. L. (2023). Who supports# MeToo and the Kavanaugh confirmation? Exploring tolerance for sexual harassment among young adults. Journal of Social Issues.

[B56-behavsci-15-00061] Holmqvist Gattario K., Lunde C. (2023). What have we learned about sexual harassment among young people? Concluding reflections. Journal of Social Issues.

[B57-behavsci-15-00061] Hong J. S., Espelage D. L. (2012). A review of research on bullying and peer victimization in school: An ecological system analysis. Aggression and Violent Behavior.

[B58-behavsci-15-00061] Hong J. S., Garbarino J. (2012). Risk and protective factors for homophobic bullying in schools: An application of the social–ecological framework. Educational Psychology Review.

[B59-behavsci-15-00061] Horn S. S., Poteat V. P. (2023). Developmental changes in young people’s evaluations of sexual harassment. Journal of Social Issues.

[B60-behavsci-15-00061] Huang F. L., Cornell D. G. (2019). School teasing and bullying after the presidential election. Educational Researcher.

[B61-behavsci-15-00061] Humphrey T., Vaillancourt T. (2020). Longitudinal relations between bullying perpetration, sexual harassment, homophobic taunting, and dating violence: Evidence of heterotypic continuity. Journal of Youth and Adolescence.

[B62-behavsci-15-00061] Interactive H., Gay L., Network S. E. (2005). From teasing to torment: School climate in America. New York: Gay, lesbian, and straight education network.

[B63-behavsci-15-00061] Jiménez-Barbero J. A., Ruiz-Hernández J. A., Llor-Zaragoza L., Pérez-García M., Llor-Esteban B. (2016). Effectiveness of anti-bullying school programs: A meta-analysis. Children and Youth Services Review.

[B64-behavsci-15-00061] Johns M. M., Lowry R., Haderxhanaj L. T., Rasberry C. N., Robin L., Scales L., Stone D., Suarez N. A. (2020). Trends in violence victimization and suicide risk by sexual identity among high school students—Youth Risk Behavior Survey, United States, 2015–2019. MMWR Supplements.

[B65-behavsci-15-00061] Kaatz T., Wang K., Burr R., Kemp J., Gbondo-Tugbawa K., Holmes W., Simon D. (2024). 2019–20 school survey on crime and safety: Public-use data file user’s manual (NCES 2024-054). National Center for Education Statistics, Institute of Education Sciences, U.S. Department of Education.

[B66-behavsci-15-00061] Kaltiala R., Ellonen N. (2022). Transgender identity and experiences of sexual harassment in adolescence. Child Abuse Review.

[B67-behavsci-15-00061] Kaltiala-Heino R., Frojd S., Marttunen M. (2016). Sexual harassment victimization in adolescence: Associations with family background. Child Abuse & Neglect.

[B68-behavsci-15-00061] Kendrick K., Jutengren G., Stattin H. (2012). The protective role of supportive friends against bullying perpetration and victimization. Journal of Adolescence.

[B69-behavsci-15-00061] Klebanov B., Friedman-Hauser G., Lusky-Weisrose E., Katz C. (2024). Sexual abuse of children with disabilities: Key lessons and future directions based on a scoping review. Trauma, Violence, & Abuse.

[B70-behavsci-15-00061] Kljakovic M., Hunt C. (2016). A meta-analysis of predictors of bullying and victimisation in adolescence. Journal of Adolescence.

[B71-behavsci-15-00061] Kuntsche E. N. (2004). Hostility among adolescents in Switzerland? Multivariate relations between excessive media use and forms of violence. Journal of Adolescent Health.

[B72-behavsci-15-00061] Lam S. F., Law W., Chan C. K., Wong B. P., Zhang X. (2015). A latent class growth analysis of school bullying and its social context: The self-determination theory perspective. School Psychology Quarterly.

[B73-behavsci-15-00061] Leemis R. W., Espelage D. L., Basile K. C., Mercer Kollar L. M., Davis J. P. (2019). Traditional and cyber bullying and sexual harassment: A longitudinal assessment of risk and protective factors. Aggressive Behavior.

[B74-behavsci-15-00061] Lereya S. T., Samara M., Wolke D. (2013). Parenting behavior and the risk of becoming a victim and a bully/victim: A meta analysis study. Child Abuse & Neglect.

[B75-behavsci-15-00061] López V., García-Quiroga M., Benbenishty R., González L., Squicciarini A. M., Sánchez P. (2020). Sexual harassment by peers in Chilean schools. Child Abuse & Neglect.

[B76-behavsci-15-00061] Mailhot Amborski A., Bussieres E. L., Vaillancourt-Morel M. P., Joyal C. C. (2022). Sexual violence against persons with disabilities: A meta-analysis. Trauma, Violence, & Abuse.

[B77-behavsci-15-00061] Miller E. M., Mondschein E. S. (2017). Sexual harassment and bullying: Similar, but not the same. What school officials need to know. The Clearing House: A Journal of Educational Strategies, Issues and Ideas.

[B78-behavsci-15-00061] Nansel T. R., Overpeck M. D., Pilla R. S., Ruan W. J., Simons-Morton B., Scheidt P. (2001). Bullying behaviours among U.S. youth: Prevalence and association with psychosocial adjustment. Journal of the American Medical Association.

[B79-behavsci-15-00061] National Center for Education Statistics (2024). Student bullying. The condition of education. *U.S. Department of Education, Institute of Education Sciences*.

[B80-behavsci-15-00061] O’Brien N., Smith P. K., Norman J. O. H. (2021). School factors with a focus on boarding schools. The Wiley Blackwell handbook of bullying: A comprehensive and international review of research and intervention.

[B81-behavsci-15-00061] Olson C. K., Kutner L. A., Baer L., Beresin E. V., Warner D. E., Nicholi A. M. (2009). M-Rated video games and aggressive or problem behavior among young adolescents. Applied Developmental Science.

[B82-behavsci-15-00061] Olweus D. (1993). Bullying at school: What we know and what we can do.

[B83-behavsci-15-00061] Ormerod A. J., Parisi T., DeBlaere C., Sagrestano L. M. (2023). School climate tolerant of sexual harassment is indirectly related to academic disengagement through peer sexual harassment and feeling safe in high school girls. Journal of Social Issues.

[B84-behavsci-15-00061] Patton D. U., Hong J. S., Williams A. B., Allen-Meares P. (2013). A review of research on school bullying among African American youth: An ecological systems analysis. Educational Psychology Review.

[B85-behavsci-15-00061] Pellegrini A. D. (2001). A longitudinal study of heterosexual relationships, aggression, and sexual harassment during the transition from primary school through middle school. Journal of Applied Developmental Psychology.

[B86-behavsci-15-00061] Pellegrini A. D. (2002). Bullying, victimization, and sexual harassment during the transition to middle school. Educational Psychologist.

[B87-behavsci-15-00061] Pepler D. J., Craig W. M., Connolly J. A., Yuile A., McMaster L., Jiang D. (2006). A developmental perspective on bullying. Aggressive Behavior: Official Journal of the International Society for Research on Aggression.

[B88-behavsci-15-00061] Peris C. A. (2020). Sexual and physical abuse of students with disabilities: The legal responsibility to provide a preventative and inclusive sexuality curriculum in Canadian schools. Education & Law Journal.

[B89-behavsci-15-00061] Peter C. R., Tasker T. B., Horn S. S. (2016). Adolescents’ beliefs about harm, wrongness, and school policies as predictors of sexual and gender-based harassment. Psychology of Sexual Orientation and Gender Diversity.

[B90-behavsci-15-00061] Phoenix A., Frosh S., Pattman R. (2003). Producing contradictory masculine subject positions: Narratives of threat, homophobia, and bullying in 11–14 year old boys. Journal of Social Issues.

[B91-behavsci-15-00061] Pouwels J. L., Garandeau C. F., Smith P. K., Norman J. O. H. (2021). The role of the peer group and classroom factors in bullying behavior. The Wiley Blackwell handbook of bullying: A comprehensive and international review of research and intervention.

[B92-behavsci-15-00061] Rolfe S. M., Schroeder R. D. (2020). “Sticks and stones may break my bones, but words will never hurt me”: Verbal sexual harassment among middle school students. Journal of Interpersonal Violence.

[B93-behavsci-15-00061] Rose C. A., Gage N. A. (2017). Exploring the involvement of bullying among students with disabilities over time. Exceptional Children.

[B94-behavsci-15-00061] Rose C. A., Gage N., Mirielli L. G., Graves K. A., Katsiyannis A., Gage N., Rapa L. K., Whitford D. K., Katsiyannis A. (2022). Bullying victimization and perpetration. Disproportionality and social justice in education.

[B95-behavsci-15-00061] Russell S. T., Kosciw J., Horn S., Saewyc E. (2010). Safe schools policy for LGBTQ students and commentaries. Social Policy Report.

[B96-behavsci-15-00061] Sapouna M., De Amicis L., Vezzali L. (2023). Bullying victimization due to racial, ethnic, citizenship and/or religious status: A systematic review. Adolescent Research Review.

[B97-behavsci-15-00061] Schwartz D., Proctor L. J. (2000). Community violence exposure and children’s social adjustment in the school peer group: The mediating roles of emotion regulation and social cognition. Journal of Consulting and Clinical Psychology.

[B98-behavsci-15-00061] Schwartz D., Ryjova Y., Fritz H., Kelleghan A., Smith P. K., Norman J. O. H. (2021). Communities and neighborhoods as contexts that influence the bully/victim dynamic. The Wiley Blackwell handbook of bullying: A comprehensive and international review of research and intervention.

[B99-behavsci-15-00061] Skoog T., Kapetanovic S. (2023). The intertwined evolution of sexual harassment victimization and emotional problems among young people. Journal of Social Issues.

[B100-behavsci-15-00061] Skoog T., Lunde C., Gattario K. H. (2023). Special issue introduction: Sexual harassment among young people. Journal of Social Issues.

[B101-behavsci-15-00061] Smith D. M., Johns N. E., Raj A. (2022). Do sexual minorities face greater risk for sexual harassment, ever and at school, in adolescence? Findings from a 2019 cross-sectional study of US adults. Journal of Interpersonal Violence.

[B102-behavsci-15-00061] Smokowski P. R., Evans C. B. (2019). Bullying and victimization across the lifespan.

[B103-behavsci-15-00061] Son E., Parish S. L., Peterson N. A. (2012). National prevalence of peer victimization among young children with disabilities in the United States. Children and Youth Services Review.

[B104-behavsci-15-00061] Stein N. (2003). Bullying or sexual harassment the missing discourse of rights in an era of zero tolerance. Arizona Law Review.

[B105-behavsci-15-00061] Stuart-Cassel V., Bell A., Springer J. F. (2011). Analysis of state bullying laws and policies. *Office of Planning, Evaluation and Policy Development, US Department of Education*.

[B106-behavsci-15-00061] Symes W., Humphrey N. (2010). Peer-group indicators of social inclusion among pupils with autistic spectrum disorders (ASD) in mainstream secondary schools: A comparative study. School Psychology International.

[B107-behavsci-15-00061] Tillyer M. S., Wilcox P., Gialopsos B. M. (2010). Adolescent school-based sexual victimization: Exploring the role of opportunity in a gender-specific multilevel analysis. Journal of Criminal Justice.

[B108-behavsci-15-00061] Ttofi M. M., Bowes L., Farrington D. P., Lösel F. (2014). Protective factors interrupting the continuity from school bullying to later internalizing and externalizing problems: A systematic review of prospective longitudinal studies. Journal of School Violence.

[B109-behavsci-15-00061] Ttofi M. M., Farrington D. P. (2011). Effectiveness of school-based programs to reduce bullying: A systematic and meta-analytic review. Journal of Experimental Criminology.

[B110-behavsci-15-00061] Turner-Moore R., Milnes K., Gough B. (2022). Bullying in five European countries: Evidence for bringing gendered phenomena under the umbrella of ‘sexual bullying’ in research and practice. Sex Roles.

[B111-behavsci-15-00061] Twyman K. A., Saylor C. F., Saia D., Macias M. M., Taylor L. A., Spratt E. (2010). Bullying and ostracism experiences in children with special health care needs. Journal of Developmental & Behavioral Pediatrics.

[B112-behavsci-15-00061] U.S. Department of Education, Office for Civil Rights (2008). Sexual harassment: It’s not academic. *U.S. Department of Education, Office for Civil Rights*.

[B113-behavsci-15-00061] Valik A., Holmqvist Gattario K., Lunde C., Skoog T. (2023). PSH-C: A measure of peer sexual harassment among children. Journal of Social Issues.

[B114-behavsci-15-00061] Vassallo S., Edwards B., Renda J., Olsson C. A. (2014). Bullying in early adolescence and antisocial behavior and depression six years later: What are the protective factors?. Journal of School Violence.

[B115-behavsci-15-00061] Waasdorp T. E., Fu R., Perepezko A. L., Bradshaw C. P. (2021). The role of bullying-related policies: Understanding how school staff respond to bullying situations. European Journal of Developmental Psychology.

[B116-behavsci-15-00061] Wang J., Iannotti R. J., Nansel T. R. (2009). School bullying among adolescents in the United States: Physical, verbal, relational, and cyber. Journal of Adolescent Health.

[B117-behavsci-15-00061] Williams K. R., Guerra N. G. (2007). Prevalence and predictors of internet bullying. Journal of Adolescent Health.

[B118-behavsci-15-00061] Wilmot J. M., Migliarini V., Ancy Annamma S. (2021). Policy as punishment and distraction: The double helix of racialized sexual harassment of Black girls. Educational Policy.

[B119-behavsci-15-00061] Yang S. C. (2012). Paths to bullying in online gaming: The effects of gender, preference for playing violent games, hostility, and aggressive behavior on bullying. Journal of Educational Computing Research.

[B120-behavsci-15-00061] Zablotsky B., Boswell K., Smith C. (2012). An evaluation of school involvement and satisfaction of parents of children with autism spectrum disorders. American Journal on Intellectual and Developmental Disabilities.

[B121-behavsci-15-00061] Zimmerman F. J., Glew G. M., Christakis D. A., Katon W. (2005). Early cognitive stimulation, emotional support, and television watching as predictors of subsequent bullying among grade-school children. Archives of Pediatrics & Adolescent Medicine.

